# Pathophysiology of Crohn’s disease inflammation and recurrence

**DOI:** 10.1186/s13062-020-00280-5

**Published:** 2020-11-07

**Authors:** L. Petagna, A. Antonelli, C. Ganini, V. Bellato, M. Campanelli, A. Divizia, C. Efrati, M. Franceschilli, A. M. Guida, S. Ingallinella, F. Montagnese, B. Sensi, L. Siragusa, G. S. Sica

**Affiliations:** 1 Department of Surgical Science, University Tor Vergata, Viale Oxford 81, 00133 Rome, Italy; 2 Torvergata Oncoscience Research Centre of Excellence, TOR, Department of Experimental Medicine, University Tor Vergata, Rome, Italy; 3 Ospedale Israelitico, Department of Gastroenterology, Rome, Italy; 4 Nuovo Ospedale dei Castelli, Endoscopy Unit, Rome, Italy

**Keywords:** Crohn’s disease, Intestinal inflammatory diseases, Crohn’s disease recurrence, Surgery

## Abstract

Chron’s Disease is a chronic inflammatory intestinal disease, first described at the beginning of the last century. The disease is characterized by the alternation of periods of flares and remissions influenced by a complex pathogenesis in which inflammation plays a key role. Crohn’s disease evolution is mediated by a complex alteration of the inflammatory response which is characterized by alterations of the innate immunity of the intestinal mucosa barrier together with a remodeling of the extracellular matrix through the expression of metalloproteins and increased adhesion molecules expression, such as MAcCAM-1. This reshaped microenvironment enhances leucocytes migration in the sites of inflammation, promoting a T_H_1 response, through the production of cytokines such as IL-12 and TNF-α. IL-12 itself and IL-23 have been targeted for the medical treatment of CD. Giving the limited success of medical therapies, the treatment of the disease is invariably surgical. This review will highlight the role of inflammation in CD and describe the surgical approaches for the prevention of the almost inevitable recurrence.

## Background

Crohn’s Disease (CD) is a chronic inflammatory intestinal disease, first described as regional ileitis by Crohn, Ginzburg and Oppenheimer in a case series presented at American Medical Association annual meeting in 1932 [1]. CD inflammation interests the whole intestine, being the most frequently affected part the distal ileum. Patients with CD experience periods of flares and periods of remissions during their disease course. Pathogenesis results from the interactions of environmental factors, immune system, susceptibility genes and host’s microbiome changes, leading to disruption of the intestinal mucosa. The role of inflammatory cells in maintaining an active disease is well known and most of the therapies aim to stop the cascade of inflammatory and pro-inflammatory cytokines.

**Fig. 1:**
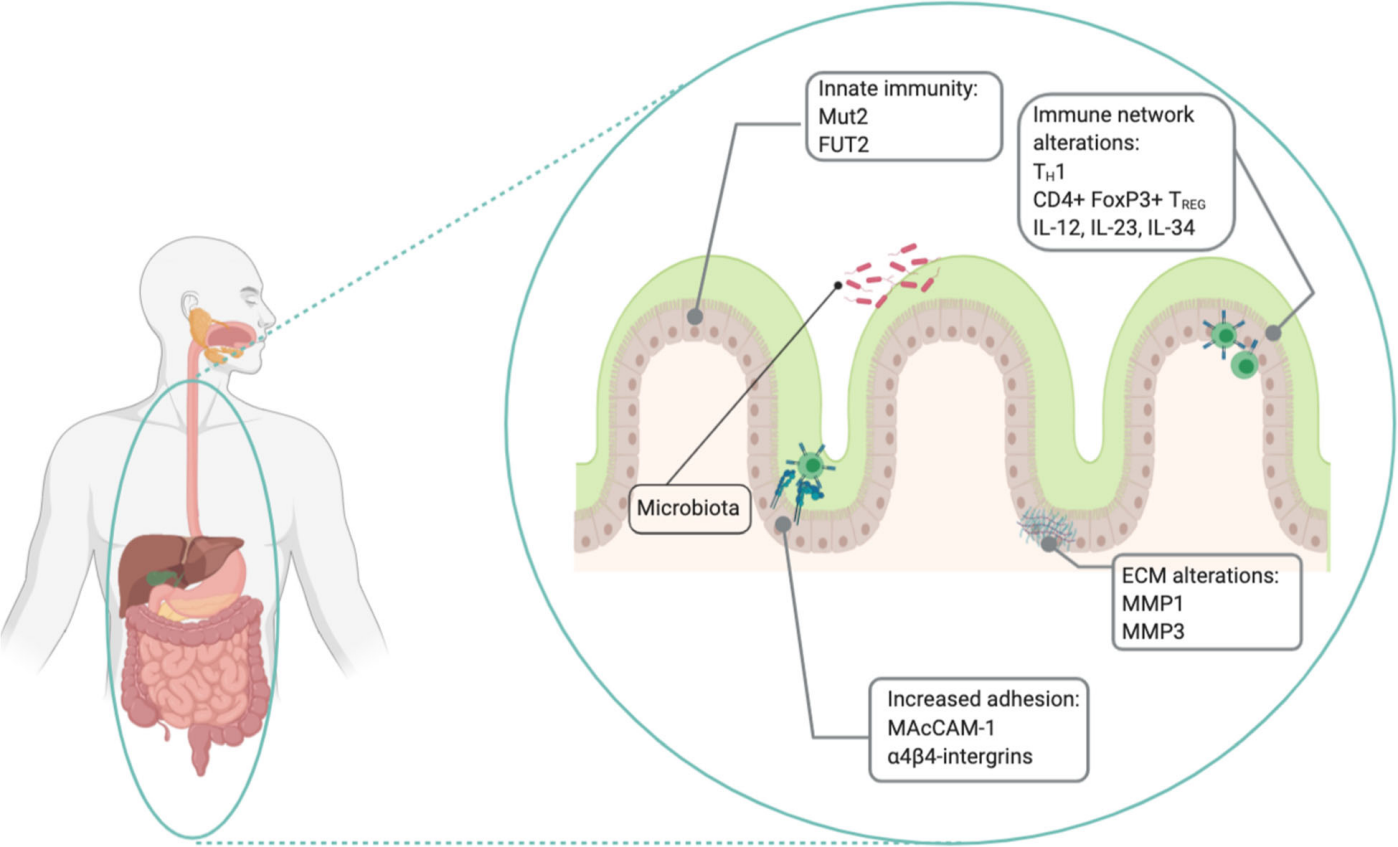
Immuno-mediated pathogenesis of Crohn’s disease. Crohn’s disease is a multifactorial pathology in which a major role is played by alterations at the level of immunity and inflammation. Innate immunity is involved in terms of defects in the mucous barrier (Mut2 and FUT2 genes) while adaptive immunity relies on a T_H_1 lymphocitic response and T_REG_ cells mediated by cytokines like TNF-α, IL-12, IL-34 and IL-23. The increased migration to the sites of inflammation is also determined by a reshaping of the extra cellular matrix through the action of metalloproteins (MMP-1 and MMP-3) and the overexpression of adhesion molecules such as MAcCAM-1 and integrin α4β4. Finally, also the host pathogen interaction between the intestinal epithelium and the microbiota has been linked to the evolution of the disease. Picture Created with BioRender.com

**Fig. 2:**
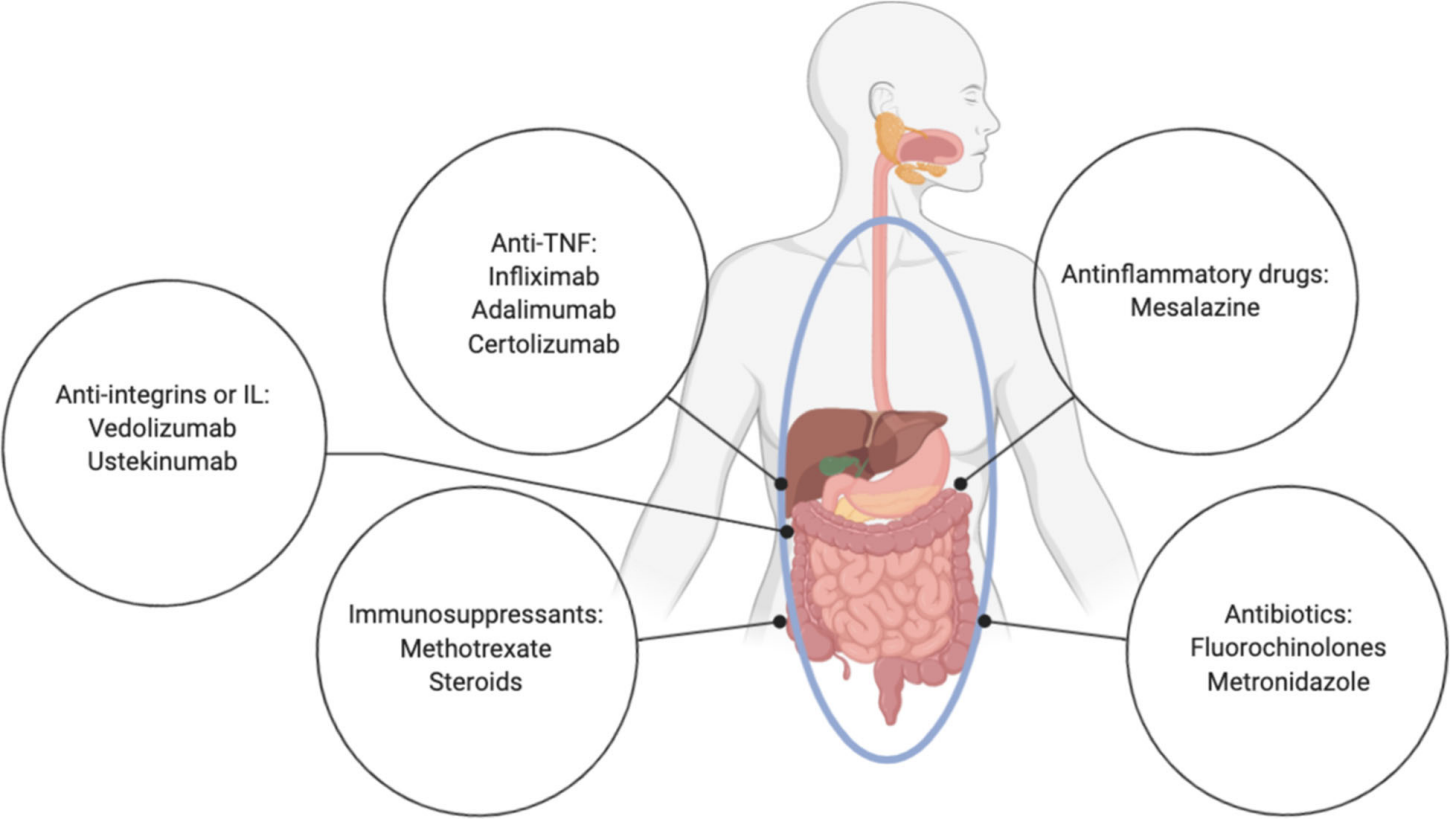
Medical treatments of Crohn’s disease. Many medical options for the treatment of Crohn’s disease are available, but still not resolutive. Among them, anti-inflammatory drugs as mesalazine, antibiotics such as fluorochinolones and metronidazole and immunosuppressants (methotrexate). More targeted treatment options are directed towards TNF-α (Infliximab, Adalimumab, Certolizumab) or against integrins (Vedolimumab) and interleukins IL-12 and IL-23 (Ustekinumab). Picture created with BioRender.com

Treatment of CD is multidisciplinary: medical treatment is focused on mucosal healing and symptoms reduction; surgery maintains a key-role in treating complications such as stenosis, perforations, fistulas and abscesses. Surgical recurrence is known to afflict over 80% of the operated patients [2, 3]. Multiple surgical strategies have been investigated to improve outcome. Introduction of laparoscopic techniques has permitted several improvements but failed in reducing recurrences; other surgical techniques are currently under evaluation in view to retard or prevent the ineluctable recurrence but the surgical cure for CD is yet to be discovered. This review is focused on CD inflammation and will discuss potential strategies to prevent recurrences, such as novel surgical approaches.

## Pathogenesis and inflammation

Crohn’s disease pathogenesis is based on tissue inflammation, caused by an unrestrainable immune response against luminal bacterial antigens (Fig. 1). Immune cells like CD4 T-Cells, CD8 T-Cells, B-Cells, CD14 monocytes and natural killers, are involved in this process as they infiltrate the gut of CD patients. Part of the immune-mediated susceptivity to CD resides in some innate mechanisms of defense form infectious diseases and the intestinal mucus secretion is part of those. It has been shown that variants of the Muc2 gene reducing mucus production are associated to CD in a mouse model. Moreover, molecules that are mediating bacterial adhesion have been correlated to the disease. This is the case of FUT2, which encodes for the fucosyltransferase enzyme, responsible for the secretion of soluble forms of the ABO antigens. People harboring a FUT2 variants decreasing the secretion of the antigens, have an altered interaction with bacteria and are more prone to developing CD [4]. The pathogenesis is also sustained by the interaction of these cells with integrins, adhesion molecules and multiple chemokines, responsible for the production of elevated levels of inflammatory cytokines, representing the target of immune and non-immune cells and the promotion of mucosal inflammation. As such, among many adhesion molecules, some evidences on the involvement of the leucocyte MAcCAM-1, receptor for the α4β4 integrin, seems to play a crucial role. Together with leucocyte adhesion, the role of the extracellular matrix on their activation has been explored. Proteins like CD44 and CD26 where shown to play a role as well as metalloproteins (MMP), being MMP1 and MMP3 abundant in the granular tissue close to CD sites of inflammation, therefore responsible for leucocytes activation [5–8]. In the mucosa of CD patients, a dysregulation of various components of the immune system is invariably found. The most pronounced alteration is the hyperactivity of T cells with excessive production of cytokines, between which IL-12 and IFN-γ, promoting a T_H_1 lymphocytic phenotype, opposed to the T_H_2 one, correlating to ulcerative colitis. Moreover, TNF-α production has also been demonstrated to increase the number of CD4+ FoxP3+ T_REG_ cells, especially in the mucosa of children affected by CD [9]. The inhibition of the effector cytokines, like TNF-α**,** attenuates the detrimental effects in subsets of CD patients. Furthermore, the expression of the interleukins, a subgroup of cytokines implicated in the enhanced or inhibition of other cytokines in many different regulatory pathways such as maturation, growth and responsiveness of immune cells population, is to be considered anomalous in CD patients [10]. Further analysis of T cell subsets has revealed the presence of T_H_1 and T_H_17 cells in CD, whereas the cytokines considered more involved are TNF, IL12 and IL23. Apart from the cited cytokines, IL-34 has also been associated to IBD and CD in particular. IL34 expression is more pronounced in the areas of active inflammation, especially in CD, and seems to induce TNF-α and IL6 expression through a ERK-mediated mechanism. Moreover, IL-34 has been described as an inducer of CCL20 through the interaction with its receptor the M-CSFR1, abundantly expressed in the inflamed colonic epithelium but not in the healthy controls. On the contrary, IL-25 inversely correlates to the inflammatory state of the patients with IBD, being reduced in CD patients as opposing to healthy subject, and being reduced in the affected areas of the colon if compared to the surrounding adjacent normal tissue from the same subject. Among all the possible interleukins associated to CD pathogenesis, IL-12 and IL-23 represent the target of still inadequate therapies because of potential side effects, such as increased risk for infection, and the blockade of specific immunological targets, capable of induction of alternative signaling or homing pathways. The latter mechanism may also partially explain the frequent lack of response to therapy with biologics such as Infliximab (Remicade©), a chimeric monoclonal antibody used to treat autoimmune diseases, that works by binding to TNF-α causing the reduction of IL-34 expression, implicated in monocyte and macrophage differentiation, survival and function [11–15].

Although T-Cells are the main effector lymphocytes in intestinal tissue inflammation, also humoral immune system plays a crucial role. Plasma cells differentiation indeed is promoted by CD4 T-Cells, through a mechanism that is firmly dependent on IL-2, overproduced in CD patient’s gut. IL-21 converts naive B-Cells into B-Cells expressing granzyme-B: it possesses a cytotoxic activity on the intestinal mucosa and perpetuates the epithelial damage. These proofs indicate that an altered balance between effector and counter regulatory factors is probably involved in the sustainment of the tissue-damaging immune response in CD [16].

Gut microbiota also plays a recognized role in designing the inflammatory response in IBD and especially in CD. There is growing evidences that some microbial gene products can influence gene expressions in the host [17–19]. The complex network arising from this assumption is referred to as the microbial-associated molecular pattern (MAMP) which is sensed by toll-like receptors on immune cells, contributing to their activation in the context of the chronic inflammation [20]. Microbiome moreover represent a source of potential pathogenic inputs that can be approached through the methods used in the omics era, such as metagenomics studies, also impacting on our knowledge on geographical variations on the clinical manifestation of the disease [21–24]. The inflammation is generally transmural and, on pathology examination, granulomas may be identified on biopsies, with a discontinuous distribution along the longitudinal axis. This inflammatory process often leads to irreversible tissue damage in the form of intestinal stenosis or fistulas, inflammatory masses or intra-abdominal abscesses. Patients can develop one or more of these disease behavior and they often tend to evolve from inflammatory to penetrating or stricturing disease [25].

## Medical management

Medical management (Fig. 2) should be tailored based on various factors such as disease severity, subtype, behavior and location [26]. Moreover, it is important to consider other factors such as age at diagnosis, extension of the lesions and extra-intestinal manifestations [27]. As matter of fact, none of the drugs used in the treatment of CD has been demonstrated to be curative or completely safe.

Mesalazine, which belong to the 5-ASA compounds category, has been evaluated in many studies and it has never shown to definitely induce or maintain remission in CD. Its benefits are related to its safety outline [28].

Antibiotics are principally recommended to treat septic complications; like mesalazine, they do not show a real efficacy in the treatment of CD, except in the short-term treatment of perianal fistula in association with anti-TNF. Most frequently used agents are fluoroquinolone and metronidazole [29].

Systemic corticosteroids show a fast onset of action and are indicated to induce remission. Unfortunately, steroid dependency or steroid resistance can jeopardize their use that is often accompanied to a wide range of side effects like obesity, hypertension, glaucoma, cataracts and adrenal insufficiency [30].

Another major part is played by immunosuppressant like thiopurines and methotrexate: thiopurines are used to maintain remission in moderate CD, usually in combination with steroids. Before starting thiopurines treatment, it is mandatory to assess TPMT (Thiopurine S-methyltransferase) activity, crucial for their metabolism [31]. Methotrexate may be considered as an option for steroid-dependent patients. Included in the side effects are hepatotoxicity and more rarely myelosuppression; they are prohibited during pregnancy because teratogenic and abortifacient [32].

Anti-TNF drugs are considered the most powerful tools to treat moderate and severe form of CD, alone or in association with immunomodulators to obtain and maintain remission. The most frequently used anti-TNF are: Infliximab (Remicade©), a chimeric antibody that is administered intravenously; Adalimumab (Humira©), a fully humanized monoclonal antibody administered subcutaneously; Certolizumab (Cimzia©), a FaB antibody fragment of humanized anti-TNF molecule [33].

More recently super-selective target monoclonal antibodies have been developed, directed against a specific pattern of inflammation. In this class there are Vedolizumab (Entyvio©), that targets the adhesion molecular inhibiting leukocyte migration [34], and interleukin-inhibitors like Ustekinumab (Stelara©), a fully humanized monoclonal antibody targeting the p-40 subunit of IL-12 and IL-23 [35].

## Surgical management and CD recurrence

When CD was described for the first time, therapy was exclusively surgical. Since the beginning of CD surgery experience, there was no consensus on the optimal procedure. At the Mount Sinai Hospital in New York, Dr. Berg was the surgeon who operated fourteen patients presented by Crohn. The “Berg” operation, also known as “Mount Sinai” operation, implied exclusion bypass of the ileocecal region, transecting small bowel proximal to the diseased ileum, over sewing distal ileum, and anastomosing the proximal ileal end into the mid-transverse colon [36, 37]. In fact, this was a staged management, being the second planned step the resection of the diseased bowel. Performing more and more cases, Mount Sinai surgeons noted that during the second stage of surgery, the bypassed bowel seemed to have “healed” in several cases. Starting from this observation and as patients manifested clinical improvements, they decided to omit the planned second procedure. Obviously, a great debate about this procedure and all early and late risks linked to it arose: blow out of the blind end, reactivation of CD in the excluded segment with abdominal pain and infections, deprivation of a large portion of the colon for water absorption. Eventually, bypass procedure was abandoned due to findings of adenocarcinoma occurring in the excluded segment [19, 38–40]. Surgical resection of the diseased bowel emerged as the procedure of choice for most patients with CD of the terminal ileum or with ileo-colitis, including complicated cases [41]. In tandem, advances in perioperative care, such as nutritional improvement, anesthesia and fluid and electrolyte management, guaranteed CD surgery improvements and safety. At this point physician focused on the amount of resection. In fact, it was commonly accepted practice for surgeons to resect all macroscopically involved intestine with large resection margins. Consciousness of CD as a pan-enteric affection and, above all, the evidence of the inevitable recurrence and possible development of short bowel syndrome due to repetitive surgery, suggested the adoption of a conservative policy, avoiding wide resections [42]. For this reason, in order to avoid short bowel syndrome - in particular in those scenarios characterized by extensive jejunal-ileitis with fibrotic stenosing segments scattered along the diseased intestine - Lee from Oxford, in 198, reported another advancement in surgical management. Lee was inspired by the work of Indian surgeons on the management of tuberculous strictures: they observed that small bowel preservation could be achieved in patients with multiple tuberculous strictures by strictureplasty [43]. Lee applied strictureplasty to the short intestinal strictures of Crohn’s disease and from that moment, with a great variability on techniques according to the different situations, strictureplasty assumed a fundamental role for CD surgeons [44, 45].

Different strictureplasty techniques have been described. Heineke-Mikulicz strictureplasty consists of a longitudinal enterotomy closed in a transverse direction and it is best applied to stricture up to 7 cm in length [46]. Finney strictureplasty is used for longer stenosis, up to 10–20 cm: after an antimesenteric longitudinal incision, the opened bowel segment is bent into a U shape and posterior and anterior layers are close with continuous absorbable suture [47]. Michelassi strictureplasty is indicated for the treatment of multiple strictures, interesting up to 90 cm long bowel segment; in this case, a segment of diseased bowel is anastomosed to a non-affected segment of intestine [48].

Interestingly this technique has shown to induce remission in the diseased part. The mechanism is still unknown, however there seems to be a process in CD whereby obstruction is responsible for the pathogenesis of many complications [49]. This technique allows the mitigation of fecal stasis, which may play a central role in postoperative mucosal healing, modifying the microbial-mucosal interaction. Possibly, the resolution of chronic obstruction may interrupt the cascade of events causing active disease [50–52].

Another cornerstone in CD surgery was represented by the advent of minimally invasive surgery. Feasibility and safety of laparoscopic ileo-cecal resection has been assessed and it was found not inferior in terms of outcomes when compared to open surgery [53–55]. Nowadays, laparoscopy is largely accepted as the first line approach for CD, in the presence of adequate expertise [56]. Laparoscopy demonstrated advantages in terms of cosmetics and postoperative recovery and assured some long-term advantages, including fewer incisional hernias, fewer adhesions and a significant impact on female fertility [57, 58]; unfortunately, no clear differences on time to recurrence was found.

From a surgical point of view CD recurrence should be considered as an inevitable consequence.

The same factors that underline the pathogenesis of CD at its first stages are thought to be responsible for post-operative recurrence (POR) setting, being the result of interplay of microbial, environmental, immunological and genetic variables [59]. Within this contest, microbial flora role seems to be linked to the fecal stream, as demonstrated by Rutgeerts et al. [60, 61] investigating the rapid recurrence of microscopic inflammation in the mucosa of excluded ileum when newly interested by fecal content.

The term post-operative recurrence is used to define the appearance of new lesions after bowel resection. Active surveillance for an early diagnosis is considered mandatory. Rutgeerts endoscopic index is possibly the most widely used scoring system to detect recurrent lesions [62, 63]. Previous studies demonstrated that the lesions are located more often in or near the area of anastomosis, usually reproducing the same initial pattern of the disease, though it has been suggested that post-resection lesions should be considered new. The presence of microscopic lesion, detected during endoscopic examinations 1 year after surgery, reinforces their role as precursors of POR. Timing of endoscopic surveillance has been discussed taking into account available evidence; recommendation is to perform endoscopic examination after 6 months from surgery or within the first year [64, 65]. Other techniques have been investigated to assess POR [66]; in particular, there is a great interest in non-invasive techniques such as ultrasonography (US). Among several emerging US technique, Small Intestine Contrast Ultrasonography (SICUS) resulted more sensitive in detecting small bowel lesions in CD patients [67]. SICUS has been demonstrated to be comparable to ileocolonscopy, also after surgery, allowing the visualization of extra luminal lesions related to CD (bowel wall thickness, mesenteric and lymph nodes enlargement). Hence, in expert hands, SICUS could be considered a valid alternative for the follow-up and early diagnosis of POR after surgery in CD [68–71].

## POR: new discoveries and trends

Other recent interesting fields of investigation focus on the role of anastomosis configuration and the mesentery function in the pathogenesis of POR.

For what concern anastomosis, a great debate was raised regard which technique should be considered optimal, in particular between the more frequent choices: side-to-side versus end-to-end configuration and mechanic stapled versus hand sewn. In 2014 He [72] carried out a meta-analysis to compare stapled side-to-side anastomosis (SSSA) and hand sewn end-to-end anastomosis (HEEA) in terms of postoperative early and late complications and POR after ileo-colic resection for CD. The conclusion was that SSSA should be preferred because of its larger luminal diameter, thus showing lower overall incidence of complications including anastomotic leak, lower recurrence and re-operation for recurrence. In 2018, Feng [73] carried out a similar meta-analysis, looking specifically at the orientation of the anastomosis. Feng concluded that SSSA iso-peristaltic is probably the optimal anastomosis because it can significantly reduce incidence of overall postoperative complications and clinical POR. The underlying idea is that, with its wide lumen configuration, SSSA iso-peristaltic reduces recurrence by preventing early stenosis, colonic reflux and secondary ischemia. In 2018 Gajendran [74] published his series, supporting the superiority of HEEA when compared to anti-peristaltic SSSA. He also looked into the impact on quality of life and inflammatory activity. Gajendran developed an experimental animal model to provide a mechanistic explanation for his clinical findings, showing that anti-peristaltic orientation alters anatomy and physiology, creating an anti-peristaltic reservoir, which causes dysmotility and alteration in contractility. In particular, the animal model showed that this is due to the perpendicular surgical trans-section of the intestinal circular muscle layers: disruption of motility seems to lead to significant structural and functional changes with local stasis of enteric contents and local distension at the anastomotic site. Gajendran concluded that, according to his data, the restoration of physiologic intestinal function with surgical reconstruction of the bowel as an intact tube could contribute to a better outcome in CD patients. In the same year, Aaltonen’s group [75] published its series regarding risk factors for anastomotic recurrence. Aaltonen’s et al. proposed a technical variant HEEA, adding an opening of the small bowel’s anti-mesenteric border to ensure enough wide bowel lumen, describing this modified technique as a safe choice for ileo-colonic resection. Current guidelines from the American Society of Colon and Rectal Surgeons [76], states that anastomosis can be constructed as deemed most appropriate by the surgeon. ECCO guidelines [53] more recently, seems to favor SSSA, taking into account He and Feng’s meta-analysis [72, 73] and stressing the concept that a wider anastomosis will have a lower rate of clinical and surgical recurrence.

Within this context, Toru Kono developed a new anastomotic technique. The first Kono-s anastomosis’ (KSA) work was published in 2011 [77]. KSA is an anti-mesenteric functional end-to-end hand sewn anastomosis, configured so that the mesentery side is located in the center of the stump. Both stumps are sutured to create a “supporting column” to maintain the diameter and dimension of the anastomosis, preventing distortion and stenosis associated with recurrent disease at the anastomotic side, especially on the mesenteric side, which represents a *locus minoris resistentiae* and so a typical recurrence location. In 2012 Fichera [78] highlighted KSA innovations: the theoretical advantages of the complete exclusion of the mesentery, the initial site of CD POR, with a true anti-mesenteric anastomosis; lower susceptibility to mechanical distortions due to the stability provided by the “supporting column”; better preservation of blood supply and innervation, achieved by dividing the mesentery close to the bowel. Moreover, in 2015, Katsuno published his series confirming safety, feasibility and good results and highlighted the easier endoscopic access to the KSA [79]. Results from the first International Multicenter Study [80], leaded by Kono himself, were available in 2015 and the authors suggested resection and KSA for CD patients who are not candidates for anti-TNF therapy due to adverse effects, loss of efficacy or financial reasons. In 2018, Seyfried [81] and Shimada [82] published two more series and in 2020, during the ECCO Congress in Vienna, Luglio presented the results of “The SuPREMe-CD Study” [83], the first randomized clinical trial comparing KSA and SSSA in terms of endoscopic and surgical recurrence, confirming a reduction in POR when KSA is performed. Multicenter trials are still needed to further confirm these preliminary results. Indeed, the interest on anastomotic configuration is still open and recently Celentano developed a model for a V-modified side-to-side, anti-mesenteric, iso-peristaltic anastomosis in which a strictureplasty is added to the inlet and the outlet of the anastomosis. Celentano’s configuration target is the widening of the lumen of the bowel in these two critical areas, with the aim of minimizing the risk of clinical and surgical anastomotic recurrence; this is just a model ex vivo but representative of the wide interest in this field [84].

The role of the mesentery could be considered the other trending topic of the last decade. The underlying idea is that the mesentery plays a prominent role in CD pathogenesis and in recurrences. Mesentery is seen as an independent organ, interposed between the intestine and the body. From its privileged location, mesentery is responsible for the conduction of local intestinal and systemic response: it is the reservoir of various cell types, in particular inflammatory ones, contained in lymph nodes and related mediators [85]. Moreover, CD mesentery shows the pathognomonic phenomenon known as fat wrapping or creeping fat [86], consisting of a peculiar form of adipose tissue hypertrophy. Creeping fat is characterized by small adipocytes, increased in number with a specific gene expression profile, accompanied to immune cell infiltration, comprising regulatory M2 macrophages and T-cells [87]. Guedj et al. [88], thanks to in vitro experiments on resection specimens of ileum from patients operated for CD, proposed a mechanism in which mesenteric adipocyte, through their production of key chemokines in response to inflammatory/bacterial stimuli, orchestrate an immune response in CD-affected mesentery.

Between 2015 and 2016, Li published two different papers regarding this topic. In the first one [89] he focused on the contribution of the mesenteric adipose tissue, measuring the fat visceral area – assimilated to the creeping fat phenomenon – in CT scan performed before surgery; in this retrospective study also subcutaneous fat area and mesenteric fat index, defined as the ratio of visceral and subcutaneous fat were considered. As result, high fat visceral area was found to be and independent predictor of early clinical recurrence of CD POR.

In the second paper [89], Li highlighted the contribution of mesenteric nerves, vessels, lymphatics and fat mass, concluding that all these structures play a crucial role in CD pathogenesis and disease progression. He provided the basis for the *Copernican revolution* in CD pathogenesis, querying the current dominant theory in CD, based on the unidirectional, “outside-in” axis of dysbiosis, innate immunity-adaptive immunity-mesentery-body system. Emerging clinical evidence strongly suggest that the axis is bidirectional, involving also all the cited mesenteric structures, and not only endoluminal agents as already cited in the pathogenesis section.

Coffey and Rivera [90, 91] gave other further hints to remove the *veil of Maya* on the pathogenesis and the mesenteric role. They started from the assumption that topographic distribution of Crohn’s disease along the intestinal tract may have a *bodily* mesenteric basis in terms of tissue volume and thickness. As a proof, they noted that the largest mesenteric region is the ileocolic region, which happens to be also the commonest localization of CD.

In 2018 Mao [92] gave more insights on how creeping fat influences stricture formation in CD; he took into account mechanisms involved in the microenvironment at interfaces of different tissue compartments, such as creeping fat, considered as an extension of mesenteric fat beginning at the intestinal hilum, and the intestine muscularis mucosa itself. As already emphasized, in creeping fat immune and nonimmune cell types are represented and increased in number, producing mediators responsible for intestinal stricture formation. Among these cells, fat-mesenchymal cells seem to play a pivotal role because their interactions appear to be important in tissue remodeling in multiple organs, including the intestine. Hence, creeping fat abandons the old role of innocent bystander and is acknowledged as an active participant in inflammation and immunity. Mao hypothesized also that stricture formation incidence has remained unchanged because no target-therapy is available and so cells involved in this process should represent a pharmacological target.

Therefore, the awareness of the central mesentery’s role provides a Copernican revolution also in terms of clinical target. In fact, from this point of view, mesentery could be considered as an *anatomical sacrarium*: there are no valid pharmacotherapeutic modalities designed specifically to manipulate it. At present, the only means of targeting the mesentery are surgical, so there is the need for development of new strategies in this field.

Even though conservative approach to intestinal resection in CD could be considered an established dogma, in the light of the recent discovered role of mesentery, the attitude is changing. Up to the present, mesentery resection has not become standard practice. This is mainly due to technical concerns regarding risk of bleeding when manipulating tissues with significant inflammation, disease-related perforation, fistula formation, adhesions, and thickened mesentery, potentially leading to complications like hemorrhage, hematomas and other injuries. Overcoming these issues and starting from the previous considerations, Coffey et al. [93] published a series on the inclusion of mesentery in ileocolic resection for CD. Clinical findings were coherent with formulated theories: inclusion of the mesentery as part of intestinal resection is associated with reduced POR, that means improved clinical outcomes, and advanced mesenteric disease resulted to be a predictor of increased risk of POR. Moreover De Groof et al. presented a series on proctectomy in CD, demonstrated that perineal complications were more frequent after close rectal dissection than after total mesorectal excision [94]. These results suggested a pathogenic role for the mesorectal tissue in CD. In the footsteps of these assumptions, an international, multicenter, randomized controlled trial [95] is ongoing about the mesenteric excision surgery versus conservative limited resection in CD. Moreover, our group is running the PANACEA study (Pathophysiological, Nodal-based Approach for Crohn’s disease Excision), a pilot yet unpublished study, based on the belief that the majority of T-cells - especially memory T-cells – lies in lymph nodes [96–99]. Our hypothesis is that mesenteric resection, including lymph nodes, should free the organism from a great number of cells involved in intestinal inflammation. Results from this study altogether with the others, will contribute to understand which of the proposed approach will be valid in reducing POR.

## Conclusions

Crohn’s disease has been seen, in the last two decades, as a multifactorial inflammatory disease. Much is known in terms of its pathogenesis from a molecular point of view from the involvement of the mucosal mucinous barrier and the role played by variants of the Mut2 or the FUT2 genes, which alters the barrier interaction with both pathogens and harmful substances, to the complex mechanisms involving aberrant expression of adhesion molecules. The leucocytic MAcCAM-1 is a mediator of integrin dependent adhesion, which is part of the mechanism leading to migration of leucocytes in CD inflamed region. On the other front, the migration of inflammatory cells is also mediated by the alteration of the extracellular matrix, often mediated by MMP proteins, being MMP-1 and MMp-3 the most abundant in this clinical context.

The complex milieu that is created by the interaction of the inflammatory cells with the intestinal epithelium is sustained by a complex network of cytokines and chemokines, which direct T lymphocytes towards a T_H_1 response, mediated by the expression of the FoxP3 transcription factor in the T_REG_ population. Among the cytokines involved in CD pathogenesis, IL-34 and IL-25 seem to play opposing roles, the first increased in the injured tissue, the second one diminished. Other cytokines such as IL-12 and IL-23 have been demonstrated paying a key role in the inflammatory response and are some of the few current medical therapeutic targets for CD. Anyhow, considering that a consistent part of CD therapeutic approaches remains surgical. We reviewed emerging surgical approaches considered useful in preventing POR in CD patients. The mesentery seems to play a pivotal role in maintaining inflammation in intestinal CD. Many studies already confirmed mesentery-based surgery as a valid approach, with improved outcomes; ongoing randomized controlled trial should help to settle the question.

## Data Availability

Not applicable.
